# Neutrophil-Related Ratios Predict the 90-Day Outcome in Acute Ischemic Stroke Patients After Intravenous Thrombolysis

**DOI:** 10.3389/fphys.2021.670323

**Published:** 2021-07-02

**Authors:** Beibei Gao, Wenjing Pan, Xueting Hu, Honghao Huang, Junli Ren, Chenguang Yang, Xinbo Zhou, Tian Zeng, Jingyu Hu, Shengqi Li, Yufan Gao, Shunkai Zhang, Guangyong Chen

**Affiliations:** ^1^Department of Internal Medicine, The Third Affiliated Hospital of Wenzhou Medical University, Wenzhou, China; ^2^Department of Neurology, The Third Affiliated Hospital of Wenzhou Medical University, Wenzhou, China; ^3^School of the First Clinical Medical Sciences, Wenzhou Medical University, Wenzhou, China

**Keywords:** neutrophil-related ratios, neutrophil-to-eosinophil ratio, acute ischemic stroke, Modified Rankin scale, platelet-to-neutrophil ratio

## Abstract

**Background and Purpose:**

Mounting researches have illuminated that the neutrophil-related ratios were related to the prognosis of acute ischemic stroke (AIS). However, few have compared their predictive value and accuracy. To make such comparison and identify the best indicator on the 90-day outcome, we investigated biomarkers including neutrophil ratio (Nr), neutrophil count (Nc), lymphocyte (L), neutrophil-to-lymphocyte ratio (NLR), platelet (P or PLT), platelet-to-neutrophil ratio (PNR), NLR-to-platelet ratio (NLR/PLT), eosinophil (E), neutrophil-to-eosinophil ratio (NER), monocyte (M), and monocyte-to-neutrophil ratio (MNR).

**Methods:**

This retrospective study recruited 283 AIS and 872 healthy controls (HCs) receiving intravenous thrombolysis (IVT). Blood samples were collected after 24 h of admission before IVT. Propensity Score Matching (PSM) was used to explore whether these ratios differentiated AIS and HCs. We applied univariate and multivariate analyses to evaluate the prediction effect of these ratios separately or added in the model and figured out a clinical prediction model. To estimate the discrimination and calibration of the new models, the receiver operating characteristics (ROC) curve analysis, DeLong method, and likelihood ratio test (LR test) were utilized.

**Results:**

PSM showed that Nr, Nc, NLR, P, PNR, NLR/PLT, NER, and MNR facilitates the differentiation of the HCs and AIS. Among the eight biomarkers, PNR and MNR could differentiate the 90-day outcome, and it was found out that PNR performed better. Univariate regression analysis demonstrated that PNR was the only independent predictor which needs no adjustment. Besides, the multivariate regression analysis, Delong method, and LR test indicated that among the neutrophil-related ratios, NLR, PNR, NLR/PLT, NER, and MNR exerted little influence on the discrimination but could enhance the calibration of the base model, and NER proved to work best.

**Conclusion:**

Low PNR was the best indicator among the neutrophil-related ratios tin predicting a poor 90-day outcome of AIS patients. Moreover, high NER performed best when predicting the 90-day outcome to improve the calibration of the base model.

## Introduction

The world is facing an epidemic of stroke. Despite the stable incidence rates and declining mortality rates, the past two decades has seen the number of incident strokes, prevalent stroke survivors, disability-adjusted life-years lost due to stroke, and stroke-related deaths increasing (Hankey, 2017). Acute ischemic stroke (AIS), a common type of stroke, can be currently treated using intravenous or intra-arterial recombinant tissue plasminogen activator (r-tPA) or mechanical endovascular therapies. However, there can be inherent risks lying in the process of r-tPA thrombolysis when benefiting eligible patients. Therefore, it is significant to find biomarkers that can predict the prognosis of AIS patients.

Leukocytes and their subtypes, as the commonly used inflammatory markers in clinical practice, are considered to be associated with infarct volume, infarct severity, and adverse outcomes (Chen et al., 2017). In addition, peripheral blood cell ratio has been put forward in many studies as a novel biomarker for predicting stroke with a crucial clinical value. A flood of neutrophil-related ratios in AIS prognosis of outcome have occurred. However, few studies compared them.

Moreover, studies on the comparison of the neutrophil-related ratios in existence for AIS prognosis are rare. Therefore, in order to compare the predictive value of the neutrophil-related ratios and find the best indicator of the 90-day outcome, our study retrospectively analyzed 283 AIS patients and 872 healthy controls (HCs) to explore the relationship between several neutrophil-related ratios and AIS 3-month outcome, and to ascertain the most effective one.

## Materials and Methods

### Data Availability

The data that support the findings of this study are available from the corresponding author on reasonable request.

### Study Population

**FIGURE 1:**
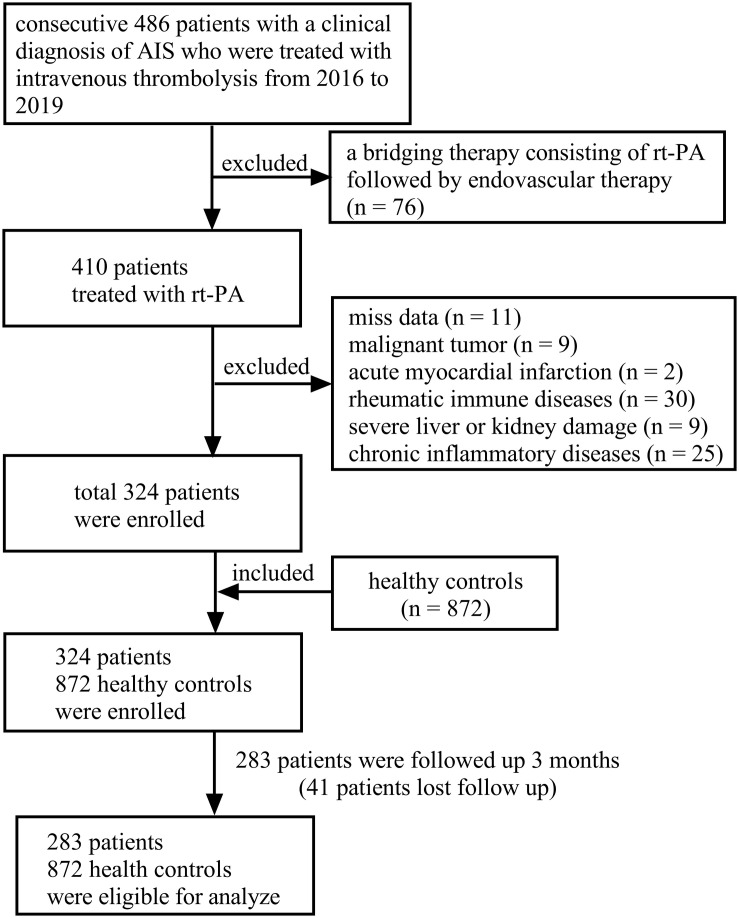
Flow diagram showing the patient selection process.

The detailed selection criteria of the study subjects were demonstrated in Figure 1. A total of 283 AIS patients who were treated with intravenous r-tPA from January 2016 to December 2019 in the Third Affiliated Hospital of Wenzhou Medical University and 872 HCs were evaluated in this retrospective study. Patients were excluded if they have (1) a bridging therapy; (2) no full baseline data; (3) a malignant tumor; (4) acute myocardial infraction; (5) rheumatic immune diseases; (6) severe liver or kidney damage; and (7) chronic inflammation.

### Data Collection

Information of HCs were obtained from examination reports. The baseline information on admission was obtained from the medical records as follows: the demographic data (age, sex), medical history (hypertension, diabetes, hyperlipidemia, coronary heart disease, atrial fibrillation), and history of smoking and drinking. National Institutes of Health Stroke Scale (NIHSS) scores on admission were evaluated by experienced clinicians. At the 3-month period after onset of AIS, Modified Rankin scale (mRS) was collected by two trained physicians on phone interview, and the death time was recorded. Hemorrhagic transformation (HT) was defined as any visible hemorrhage on the brain CT within 24 h after thrombolysis. Recurrent stroke was collected by telephone contact and medical records. Blood samples were collected after 24 h of admission before intravenous thrombolysis (IVT). Neutrophil ratio (Nr), neutrophil count (Nc), lymphocyte (L), platelet (P or PLT), eosinophil (E), and monocyte (M) were determined by XT-1800i (Sysmex, Kobe, Japan). Neutrophil-to-lymphocyte ratio (NLR) was calculated as the absolute value of the ratio of neutrophils to lymphocytes, platelet-to-neutrophil ratio (PNR) was calculated as the absolute value of the ratio of platelets to neutrophils, NLR-to-platelet ratio (NLR/PLT) was calculated as the absolute value of the ratio of NLR to platelets, neutrophil-to-eosinophil ratio (NER) was calculated using neutrophil counts divided by eosinophil counts, and monocyte-to-neutrophil ratio (MNR) was calculated using monocytes to neutrophils.

### Diagnostic Criteria

Stroke severity was assessed using the NIHSS. Good recovery was defined as mRS scores ranking 0–2 while death or dependence was defined as mRS scores ranking 3–6 at 3 months after stroke. Hemorrhagic transformation (HT) was defined as any visible hemorrhage on the brain CT within 24 h after thrombolysis.

### Statistical Analysis

Statistical analyses were performed via the SPSS Statistics 25.0 software (SPSS Inc., Chicago, IL) and R version 4.0.2 (R Foundation for Statistical Computing, Vienna, Austria). Continuous variables that are consistent with the normal distribution were expressed as mean ± Standard Deviation (SD) whereas continuous variables that do not conform to the normal distribution were expressed as medians and interquartile range, while categorical variables were expressed as frequencies and percentage. The differences between the groups of continuous variables were compared by an independent sample *t*-test or Mann-Whitney *U*-test according to the normal distribution. In order to accurately compare the neutrophil-related ratios between the HCs and AIS patients, Propensity Score Matching (PSM) was used to match the baseline data between the two groups by age and sex with the match tolerance set at 0.1. Patients were divided into two groups according to the NIHSS on admission, 3-month mRS, and HT, respectively, and their baseline data were compared to investigate whether the neutrophil-related indicators were related to the severity of the illness on admission and the 3-month prognosis. Univariate and multivariate logistic analyses were performed to estimate the association between the neutrophil-related ratios and AIS outcomes. Variables with a *p* < 0.10 in the univariate analysis were entered in the model 2. In univariate analysis, it is better to relax *p*-value to 0.10 to avoid missing possible important factors. It should be noted that, the interaction among variables may lead to the results of the multivariate analyses different from that of the univariate analyses, and we should focus on the latter. The variables with a statistical significance of *p* < 0.10 in the univariate analysis and *p* < 0.05 in the multivariate model 2 were entered in model 3, which was also called as the base model.

The receiver operating characteristics (ROC) curve analysis was applied to analyze the accuracy of the models when evaluating the stroke severity on admission and prognosis for the 3-month outcome of AIS patients receiving thrombolysis. The differences in the discriminative ability were tested using the DeLong method while the calibration of models was estimated by the likelihood ratio test (LR test). Statistical significance was set at *p* < 0.05.

## Results

### Baseline Characteristics of the Study Subjects

Among all enrolled subjects, 283 were AIS patients and 872 were HCs. The characteristics of the AIS patients and HCs were displayed in Table 1. Before PSM, a higher level of Nc and lower levels of L, P, and M led to higher NLR levels [1.94 (1.44–3.11) vs. 1.44 (1.16–1.85); *p* < 0.001], lower PNR levels [46.00 (34.81–59.80) vs. 71.43 (57.65–87.98); *p* < 0.001], and lower MNR levels [0.11 (0.08–0.14) vs. 0.13 (0.10–0.16); *p* < 0.001] in the AIS patients. The NLR/PLT levels elevated as the level of NLR grew higher and P or called PLT lowered [1.07 (0.73–1.79) vs. 0.62 (0.49–0.83); *p* < 0.001]. Nc was the only component contributing to higher NER levels, since there was no statistical difference between the AIS patients and HCs in E (0.15 ± 0.15 vs. 0.16 ± 0.15, *p* = 0.256). After matching by age and sex, the PNR levels [50.09 (36.78–62.51) vs. 70.83 (54.03–89.23); *p* < 0.001] in the AIS patients were still lower than those in the HCs, while the NLR/PLT levels [1.01 (0.69–1.65) vs. 0.66 (0.49–0.90); *p* < 0.001] were higher. However, there was no statistical difference between the AIS patients and HCs in L [2.00 (1.50–2.58) vs. 2.21 (1.74–2.60), *p* = 0.444] and E (0.15 ± 0.13 vs. 0.17 ± 0.17, *p* = 0.122), which means that NLR, NER, and MNR were only affected by Nc (Table 1).

**TABLE 1:** Demographic and laboratory characteristics of the AIS patients and healthy controls before and after matching (1:1 match, caliper 0.1).

	Before propensity score matching			After propensity score matching		
Variables	Patient group (*n* = 283)	Control group (*n* = 872)	*p-*value	Patient group (*n* = 148)	Control group (*n* = 148)	*p-*value
Nr (%)	59.80 (53.20–69.20) ↑	53.40 (48.40–58.90)	<0.001^b^	59.80 (53.25–68.48) ↑	54.15 (48.53–59.28)	<0.001^b^
Nc (10^9/L)	4.10 (3.30–5.30) ↑	3.26 (2.61–4.05)	<0.001^b^	4.20 (3.30–5.20) ↑	3.39 (2.50–4.03)	<0.001^b^
L(10^9/L)	1.50 (1.10–1.90) ↓	2.24 (1.86–2.64)	0.002 ^b^	2.00 (1.50–2.58)	2.21 (1.74–2.60)	0.444^b^
NLR	1.94 (1.44–3.11) ↑	1.44 (1.16–1.85)	<0.001^b^	1.88 (1.40–3.05) ↑	1.47 (1.20–1.93)	<0.001^b^
P(10^9/L)	193.10 ± 52.52 ↓	235.43 ± 50.73	<0.001^a^	202.54 ± 48.29 ↓	228.97 ± 55.44	<0.001^a^
PNR	46.00 (34.81–59.80) ↓	71.43 (57.65–87.98)	<0.001^b^	50.09 (36.78–62.51) ↓	70.83 (54.03–89.23)	<0.001^b^
NLR/PLT*100	1.07 (0.73–1.79) ↑	0.62 (0.49–0.83)	<0.001^b^	1.01 (0.69–1.65) ↑	0.66 (0.49–0.90)	<0.001^b^
E(10^9/L)	0.15 ± 0.15	0.16 ± 0.15	0.256^a^	0.15 ± 0.13	0.17 ± 0.17	0.122^ a^
NER	35.38 (22.22–77.14) ↑	27.44 (16.24–46.44)	<0.001^b^	38.75 (20.87–81.67) ↑	28.04 (13.78–48.15)	0.003 ^b^
M (10^9/L)	0.40 (0.30–0.60) ↓	0.42 (0.34–0.52)	0.002^b^	0.40 (0.30–0.60)	0.43 (0.33–0.55)	0.310^ a^
MNR	0.11 (0.08–0.14) ↓	0.13 (0.10–0.16)	<0.001^b^	0.11 ± 0.06 ↓	0.14 ± 0.04	<0.001^a^

*^a^Independent sample t-test. ^b^Mann–Whitney U-test. Nr, neutrophil ratio; Nc, neutrophil count; L, lymphocyte; NLR, neutrophil-to-lymphocyte ratio; P, platelet; PNR, platelet-to-neutrophil ratio; NLR/PLT, NLR-to-platelet ratio; E, eosinophil; NER, neutrophil-to-eosinophil ratio; M, monocyte; MNR, monocyte-to-neutrophil ratio. The match tolerance set at 0.1.*

### PNR and MNR Distinguishing a Poor 3-Month Outcome

At the 3-month follow-up, 41 (12.65%) patients were lost and excluded from the study. The remaining 283 patients were valid for the analysis. The mean PNR was 50.00 in the independence group (mRS 0–2) and 44.43 in the death or dependence group (mRS 3–6), which indicated that the PNR levels are able to distinguish a poor 3-month outcome (*p* = 0.023). MNR, slightly inferior to PNR, could also distinguish a poor 3-month outcome as 0.12 vs. 0.11 in the independence group and death or dependence group with *p* = 0.043. Moreover, there was no significant difference between the severity in the neutrophil-related ratios and the severity of stroke on admission. What is more, the results of the neutrophil-related ratios in predicting HT did not reach a statistical significance. We could draw that both the severe AIS patients and those with a poor outcome at 3 months were older, smoked and drank less, and suffered more from atrial fibrillation (Table 2).

**TABLE 2:** Characteristics of the AIS patients on the severity of stroke on admission, outcome at 3 months, and hemorrhagic transformation according to the neutrophil-related ratios.

	Admission NIHSS			mRS in 3 months			Hemorrhagic transformation*		
	Mild stroke NIHSS 0–10	Severe stroke NIHSS > 10	*p-*value	Independence mRS 0–2	Death or dependence mRS 3–6	*p-*value	NO HT	HT	*p-*value
	(*n* = 206)	(*n* = 77)		(*n* = 196)	(*n* = 87)		(*n* = 239)	(*n* = 38)	
Nr (%)	60.66 ± 11.53	62.45 ± 11.99	0.223^a^	60.24 ± 11.32	62.94 ± 12.26	0.072^a^	60.98 ± 11.25	60.50 ± 13.36	0.813^a^
Nc (10^9/L)	4.42 ± 1.73	4.57 ± 1.76	0.500^a^	4.35 ± 1.69	4.70 ± 1.84	0.117^a^	4.47 ± 1.74	4.21 ± 1.67	0.390^a^
L (10^9/L)	2.15 ± 0.96	2.00 ± 0.84	0.228^a^	2.13 ± 0.92	2.06 ± 0.96	0.516^a^	2.12 ± 0.91	2.06 ± 1.04	0.678^a^
NLR	2.57 ± 2.00	2.76 ± 1.79	0.456^a^	1.89 (1.42–2.92)	2.14 (1.50–3.57)	0.061^b^	2.57 ± 1.84	2.73 ± 2.38	0.629^a^
P (10^9/L)	195.16 ± 51.73	187.61 ± 54.57	0.283^a^	195.30 ± 51.68	188.16 ± 54.35	0.292^a^	194.39 ± 52.27	190.87 ± 52.70	0.700^a^
PNR	49.04 ± 19.03	46.27 ± 18.95	0.277^a^	50.00 ± 19.44	44.43 ± 17.50	0.023 ^a^	48.34 ± 9.00	50.73 ± 18.77	0.471^a^
NLR/PLT *100	1.43 ± 1.28	1.60 ± 1.26	0.331^a^	1.01 (0.71–1.72)	1.20 (0.78–2.03)	0.050 ^b^	1.42 ± 1.13	1.63 ± 1.81	0.338^a^
E (10^9/L)	0.15 ± 0.16	0.14 ± 0.12	0.409^a^	0.15 ± 0.15	0.14 ± 0.14	0.659 ^a^	0.15 ± 0.16	0.13 ± 0.12	0.408^a^
NER	73.67 ± 113.55	65.29 ± 68.30	0.544^a^	35.47 (21.98–72.23)	35.00 (24.17–94.00)	0.093 ^b^	68.95 ± 94.39	88.38 ± 151.80	0.286^b^
M (10^9/L)	0.49 ± 0.21	0.47 ± 0.26	0.609^a^	0.24 ± 0.17	0.46 ± 0.18	0.211 ^a^	0.49 ± 0.23	0.46 ± 0.20	0.471^a^
MNR	0.12 ± 0.06	0.11 ± 0.57	0.312^a^	0.12 ± 0.06	0.11 ± 0.05	0.043^a^	0.12 ± 0.0.58	0.06 ± 0.01	0.617^a^
Age	66.05 ± 12.28	71.94 ± 13.49	0.001^a^	66.00 (55.00–75.75)	75.00 (64.00–82.00)	<0.001^b^	67.33 ± 12.77	69.08 ± 14.10	0.439^a^
Male (%)	138 (66.99)	42 (54.55)	0.061^b^	130 (66.33)	50 (57.47)	0.163^b^	156 (65.27)	19 (50.00)	0.088^b^
Hypertension (%)	126 (61.17)	47 (61.04)	0.985^a^	115 (58.67)	58 (66.67)	0.198^b^	148 (61.92)	22 (57.89)	0.637^a^
Diabetes (%)	39 (18.93)	13 (16.88)	0.693^a^	37 (18.88)	15 (17.24)	0.744^a^	46 (19.25)	6 (15.79)	0.614^a^
HPL (%)	22 (10.68)	9 (11.69)	0.810^a^	18 (9.18)	13 (14.64)	0.189^b^	27 (11.30)	4 (10.53)	0.889^a^
CHD (%)	10 (4.85)	7 (9.09)	0.245^b^	11 (5.61)	6 (6.90)	0.676^a^	14 (5.86)	3 (7.89)	0.628^a^
AF (%)	36 (17.48)	28 (36.36)	0.003^b^	34 (17.35)	30 (34.48)	0.004^b^	45 (18.83)	17 (44.74)	0.004^b^
Smoking (%)	91 (44.17)	21 (27.27)	0.007^b^	89 (45.41)	23 (26.44)	0.002^b^	98 (41.00)	15 (39.47)	0.859^a^
Drinking (%)	83 (40.29)	22 (28.57)	0.061^b^	83 (42.35)	22 (25.29)	0.004^b^	91 (38.08)	12 (31.58)	0.443^a^

^a^Independent sample t-test. ^b^Mann–Whitney U-test. *277 patients were enrolled for hemorrhagic transformation. NIHSS, national institute of health stroke scale; mRS, Modified Rankin Scale; HT, hemorrhagic transformation; OR, odd ratio; CHD, coronary heart disease; AF, atrial fibrillation; Nr, neutrophil ratio; HPL, hyperlipidemia; Nc, neutrophil count; L, lymphocyte; NLR, neutrophil-to-lymphocyte ratio; P, platelet; PNR, platelet-to-neutrophil ratio; NLR/PLT, NLR-to-platelet ratio; E, eosinophil; NER, neutrophil-to-eosinophil ratio; M, monocyte; MNR, monocyte-to-neutrophil ratio.

### Some Neutrophil-Related Ratios Predict the 3-Month Outcome

As for the severity of stroke on admission, univariate logistic regression showed that no ratios among the neutrophil-related ratios had a statistical significance. We selected the variables with a *p* < 0.10 in model 1 as covariates in the multivariate analysis. Age [OR = 1.031 (1.007–1.056), *p* = 0.012] and atrial fibrillation [OR = 2.421 (1.300–4.509), *p* = 0.005] still displayed significance, which were further adjusted in model 3. Therefore, age and atrial fibrillation can form a good enough base model I to predict the severity of stroke on admission.

As for the 3-month outcome, the univariate logistic regression analysis demonstrated that age, hypertension, atrial fibrillation, smoking, drinking, and NIHSS on admission was associated with a poor 3-month outcome. Among the neutrophil-related ratios, PNR was found to be the only predictive indicator that could act individually, and a lower PNR was to predict a poor 90-day outcome of AIS patients. In model 2, we chose the variables with a *p* < 0.10 in model 1 as covariates, and in this multivariate analysis, age [OR = 1.045 (1.017–1.075), *p* = 0.002] and NIHSS [OR = 1.183 (1.119–1.251), *p* < 0.001] were still significant (*p* < 0.05), which were adjusted in model 3. Therefore, age and NIHSS composed base model II. After adjusting for age and admission NIHSS scores, Nr, Nc, NLR, PNR, NLR/PLT, NER, and MNR all had a *p*-value lower than 0.05, among which, NER had the least (*p* = 0.001). Therefore, NER served as the best indicator to improve the calibration of the base model to predict the 90-day outcome, and its elevation exerts an adverse effect on the outcome.

As for HT, univariate logistic regression showed that no ratios among the neutrophil-related ratios had a statistical significance. Univariate logistic regression analysis demonstrated that sex, atrial fibrillation, and NIHSS on admission was associated with HT. We selected the variables with *p* < 0.10 in model 1 as covariates in the multivariate analysis. Atrial fibrillation [OR = 3.342 (1.579–7.074), *p* = 0.002] and NIHSS [OR = 1.073 (1.018–1.130), *p* = 0.009] still displayed significance, which were further adjusted in model 3. Therefore, NIHSS and atrial fibrillation can form a good enough base model III to predict whether patients would suffered from HT.

Whether for the stroke severity on admission or the 3-month outcome prognosis or HT, the neutrophil-related ratios revealed no alteration in models 2 and 3. Compared with model 1, model 3 demonstrated a more comprehensive result by adjusting the confounding factors, and were of more value and concision compared with model 2. Therefore, model 3 was considered the best model due to its best clinical significance (Table 3).

**TABLE 3:** Adjusted Models for prognosis at 3 months, the severity of stroke on admission, and hemorrhagic transformation.

	Variables	Admission NIHSS score (>10) OR (95% CI)	*p-*value	3-month mRS (3–6) OR (95% CI)	*p-*value	Hemorrhagic transformation OR (95% CI)	*p-*value
Model 1	Age	1.040 (1.016–1.063)	0.001	1.058 (1.034–1.084)	<0.001	1.011 (1.984–1.039)	0.438
	Sex	0.591 (0.347–1.009)	0.054	1.409 (0.830–2.390)	0.204	0.532 (0.267–1.060)	0.073
	Hypertension	0.995 (0.581–1.702)	0.985	1.750 (1.071–2.859)	0.025	0.845 (0.422–1.694)	0.636
	Diabetes	0.870 (0.436–1.735)	0.692	0.895 (0.462–1.735)	0.743	0.787 (0.311–1.993)	0.613
	Hyperlipidemia	1.107 (0.486–2.523)	0.809	1.737 (0.810–3.726)	0.156	0.924 (0.304–2.805)	0.889
	CHD	1.960 (0.718–5.348)	0.189	1.246 (0.445–3.484)	0.675	1.378 (0.377–5.039)	0.628
	AF	2.698 (1.500–4.855)	0.001	2.508 (1.409–4.462)	0.002	3.490 (1.704–7.148)	0.001
	Smoking	0.474 (0.267–0.840)	0.010	0.432 (0.248–0.751)	0.003	0.938 (0.466–1.889)	0.858
	Drinking	0.593 (0.336–1.045)	0.071	0.461 (0.263–0.807)	0.007	0.751 (0.361–1.561)	0.443
	NIHSS			1.199 (1.136–1.266)	<0.001	1.085 (1.032–1.141)	0.001
	Nr (%)	1.014 (0.992–1.037)	0.233	1.017 (0.998–1.037)	0.080	0.995 (0.967–1.027)	0.813
	Nc (10^9/L)	1.052 (0.908–1.219)	0.499	1.124 (0.998–1.267)	0.054	0.910 (0.733–1.129)	0.389
	L (10^9/L)	0.832 (0.616–1.123)	0.228	0.958 (0.751–1.222)	0.729	0.922 (0.628–1.353)	0.677
	NLR	1.051 (0.923–1.196)	0.456	1.080 (0.982–1.188)	0.112	1.042 (0.882–1.232)	0.628
	P (10^9/L)	0.997 (0.992–1.002)	0.282	0.997 (0.993–1.002)	0.219	0.999 (0.992–1.005)	0.699
	PNR	0.992 (0.978–1.006)	0.277	0.982 (0.969–0.995)	0.007	1.006 (0.989–1.024)	0.470
	NLR/PLT*100	1.101 (0.906–1.337)	0.333	1.140 (0.978–1.328)	0.094	1.123 (0.884–1.426)	0.341
	E (10^9/L)	0.438 (0.061–3.121)	0.410	0.765 (0.121–4.824)	0.776	0.304 (0.018–5.072)	0.407
	NER	0.999 (0.996–1.002)	0.545	1.002 (1.000–1.004)	0.098	1.001 (0.999–1.004)	0.293
	M (10^9/L)	0.726 (0.213–2.472)	0.608	1.072 (0.308–3.726)	0.913	0.524 (0.091–3.005)	0.468
	MNR	0.076 (0.001–11.324)	0.313	0.045 (0.000–6.197)	0.217	4.037 (0.017–952.486)	0.617
Model 2	Age	1.031 (1.007–1.056)	0.012	1.045 (1.017–1.075)	0.002	0.995 (0.967–1.027)	0.813
	Sex	0.665 (0.327–1.353)	0.261			0.484 (0.233–1.005)	0.052
	Hypertension			1.461 (0.779–2.740)	0.238		
	Diabetes						
	Hyperlipidemia						
	CHD						
	AF	2.421 (1.300–4.509)	0.005	1.361 (0.679–2.728)	0.385	3.342 (1.579–7.074)	0.002
	Smoking	0.735 (0.431–1.342)	0.431	0.740 (0.358–1.531)	0.417		
	Drinking	1.005 (0.512–1.976)	0.988	0.721 (0.350–1.485)	0.375		
	NIHSS			1.183 (1.119–1.251)	<0.001	1.073 (1.018–1.130)	0.009
	Nr (%)	1.015 (0.992–1.040)	0.202	1.029 (1.002–1.056)	0.032	0.993 (0.963–1.025)	0.663
	Nc (10^9/L)	1.096 (0.940–1.279)	0.243	1.227 (1.036–1.452)	0.018	0.932 (0.747–1.164)	0.535
	L (10^9/L)	0.870 (0.640–1.184)	0.377	0.924 (0.667–1.281)	0.637	0.981 (0.664–1.451)	0.925
	NLR	1.061 (0.926–1.217)	0.393	1.227 (1.059–1.421)	0.007	1.046 (0.876–1.248)	0.619
	P (10^9/L)	1.000 (0.995–1.006)	0.911	1.001 (0.995–1.007)	0.666	1.001 (0.994–1.008)	0.840
	PNR	0.994 (0.979–1.009)	0.411	0.983 (0.967–1.000)	0.046	1.008 (0.990–1.027)	0.395
	NLR/PLT*100	1.068 (0.869–1.313)	0.531	1.005 (1.002–1.007)	0.031	1.114 (0.861–1.441)	0.411
	E (10^9/L)	0.602 (0.079–4.604)	0.602	0.906 (0.132–6.226)	0.920	0.738 (0.041–13.20)	0.837
	NER	0.999 (0.996–1.002)	0.619	1.003 (1.000–1.005)	0.001	1.002 (0.998–1.005)	0.329
	M (10^9/L)	0.932 (0.256–3.402)	0.915	0.444 (0.102–1.922)	0.277	0.941 (0.158–5.626)	0.947
	MNR	0.042 (0.000–9.404)	0.250	0.000 (0.000–0.161)	0.013	20.227 (0.055–7409.545)	0.318
Model 3	Nr (%)	1.014 (0.991–1.038)	0.229	1.029 (1.002–1.056)	0.032	0.993 (0.963–1.025)	0.667
	Nc (10^9/L)	1.089 (0.935–1.268)	0.274	1.218 (1.034–1.435)	0.018	0.928 (0.743–1.159)	0.511
	L (10^9/L)	0.890 (0.655–1.209)	0.455	0.946 (0.685–1.309)	0.739	0.987 (0.667–1.461)	0.947
	NLR	1.057 (0.922–1.210)	0.428	1.219 (1.054–1.410)	0.008	1.044 (0.875–1.246)	0.631
	P (10^9/L)	1.001 (0.996–1.006)	0.718	1.001 (0.996–1.007)	0.635	1.002 (0.995–1.009)	0.622
	PNR	0.996 (0.982–1.011)	0.604	0.984 (0.968–1.000)	0.049	1.010 (0.992–1.029)	0.267
	NLR/PLT*100	1.052 (0.854–1.294)	0.635	1.261 (1.022–1.577)	0.031	1.097 (0.846–1.423)	0.486
	E (10^9/L)	0.397 (0.051–3.112)	0.379	0.733 (0.102–5.263)	0.758	0.421 (0.023–7.697)	0.560
	NER	1.000 (0.997–1.003)	0.786	1.005 (1.002–1.007)	0.001	1.002 (0.999–1.005)	0.285
	M (10^9/L)	0.750 (0.208–2.712)	0.661	0.440 (0.105–1.842)	0.261	0.714 (0.121–4.224)	0.710
	MNR	0.024 (0.000–5.270)	0.176	0.000 (0.000–0.146)	0.011	8.645 (0.026–2,886.793)	0.467

*Model 1 is univariate regression analysis. Model 2 for NIHSS (> 10) was adjusted for age, sex, atrial fibrillation, smoking, and drinking while for mRS (3–6) was adjusted for age, hypertension, atrial fibrillation, smoking, drinking and admission NIHSS scores, and for hemorrhagic transformation was adjusted for sex, atrial fibrillation, and NIHSS scores. Model 3 for NIHSS (> 10) was adjusted for age and atrial fibrillation while for mRS (3–6) was adjusted for age and admission NIHSS scores, and for hemorrhagic transformation was adjusted for atrial fibrillation and NIHSS scores. Abbreviations: NIHSS, national institute of health stroke scale; mRS, Modified Rankin Scale; OR, odd ratio; HT, hemorrhagic transformation; CHD, coronary heart disease; AF, atrial fibrillation; Nr, neutrophil ratio; Nc, neutrophil count; L, lymphocyte; NLR, neutrophil-to-lymphocyte ratio; P, platelet; PNR, platelet-to-neutrophil ratio; NLR/PLT, NLR-to-platelet ratio; E, eosinophil; NER, neutrophil-to-eosinophil ratio; M, monocyte; MNR, monocyte-to-neutrophil ratio.*

### Some Neutrophil-Related Ratios Improve the Models

How close a model could come to the reality can be estimated by two characteristics, the discrimination and calibration. The ROC analysis or C statistic is a frequently used assessment method to characterize the discrimination. As shown, when the neutrophil-related ratios were added in base model 1 and model 2, the *p*-value of the new models indicated a strong statistical significance at *p* < 0.001, compared to that of *p* > 0.05 using the DeLong method. Consequently, in spite of an excellent performance in ROC, the new models did not really ameliorate the base model in discrimination, nor did it worsen. Calibration or goodness of fit is often considered as one of the most essential properties in a model, reflecting the extent of model correctness when estimating risks. In this study, LR test was utilized to compare the calibration between the base models and new models. Log-likelihood ratio (LLR) and AIC (Akaike Information Criterion) were both employed to evaluate the calibration of models in logistic analysis. LLR is positively correlated with the improvement in model performance, while AIC displays a reverse effect. For the 3-month outcome, the *p*-values of Nr, Nc, NLR, PNR, NLR/PLT, NER, and MNR were all less than 0.05 in the LR test and their AIC were all smaller than the base models. Furthermore, it can be noted that both the numerator and denominator of the meaningful ratio are Nc meaningful, while the other is meaningless. Then, we took the AIC of Nc as the standard, smaller than it were NLR, NER, and MNR, among which, the AIC of NER was the smallest and presented the most notable improvement. For the severity of stroke on admission and HT, there were no models with the *p-*value smaller than 0.05 in the Delong method, and none improved the calibration in the base model (Table 4).

**TABLE 4:** Diagnostic values of the base models and when the neutrophils-related ratios are added for the severity of stroke on admission, stroke outcome at 3 months, and hemorrhagic transformation.

	C statistic			LR test			
	Estimate (95% CI)	*p*-value (ROC)	*p*-value (DeLong’s test)	LLR	LLR improvement from base model	*p*-value	AIC
**Severe stroke (NIHSS, > 10)**							
Base model I	0.667 (0.591–0.743)	<0.001	Reference	−155.87	Reference	Reference	317.74
Base model I + Nr	0.673 (0.598–0.748)	<0.001	0.554	−155.14	0.73	0.228	318.28
Base model I + Nc	0.673 (0.597–0.749)	<0.001	0.481	−155.28	0.59	0.279	318.57
Base model I + L	0.669 (0.594–0.745)	<0.001	0.717	−155.58	0.29	0.449	319.17
Base model I + NLR	0.674 (0.598–0.750)	<0.001	0.292	−155.56	0.31	0.433	319.12
Base model I + P	0.669 (0.593–0.745)	<0.001	0.552	−155.80	0.07	0.718	319.61
Base model I + PNR	0.669 (0.593–0.746)	<0.001	0.639	−155.73	0.14	0.602	319.47
Base model I + NLR/PLT*100	0.670 (0.595–0.746)	<0.001	0.451	−155.76	0.11	0.640	319.52
Base model I + E	0.680 (0.614–0.746)	<0.001	0.506	−155.45	0.42	0.358	318.89
Base model I + NER	0.666 (0.590–0.742)	<0.001	0.598	−155.83	0.04	0.783	319.66
Base model I + M	0.667 (0.592–0.743)	<0.001	0.940	−155.77	0.10	0.656	319.54
Base model I + MNR	0.669 (0.594–0.745)	<0.001	0.871	−155.06	0.81	0.203	318.12
**Death or dependence in 3 months (mRS, 3**–**6)**							
Base model II	0.805 (0.751–0.859)	<0.001	Reference	−136.48	Reference	Reference	278.96
Base model II + Nr	0.818 (0.766–0.871)	<0.001	0.186	−134.10	2.38	0.029	276.20
Base model II + Nc	0.817 (0.765–0.868)	<0.001	0.245	−133.76	2.72	0.020	275.52
Base model II + L	0.807 (0.753–0.861)	<0.001	0.342	−136.42	0.06	0.738	280.85
Base model II + NLR	0.820 (0.768–0.872)	<0.001	0.224	−132.94	3.54	0.008	273.87
Base model II + P	0.805 (0.751–0.860)	<0.001	0.842	−136.37	0.11	0.635	280.73
Base model II + PNR	0.819 (0.768–0.870)	<0.001	0.152	−134.43	2.05	0.043	276.85
Base model II + NLR/PLT*100	0.818 (0.767–0.870)	<0.001	0.208	−134.16	2.32	0.031	276.32
Base model II + E	0.807 (0.753–0.861)	<0.001	0.350	−136.43	0.05	0.755	280.86
Base model II + NER	0.827 (0.776–0.878)	<0.001	0.146	−130.87	5.61	<0.001	269.74
Base model II + M	0.810 (0.756–0.863)	<0.001	0.362	−135.79	0.69	0.241	279.58
Base model II + MNR	0.824 (0.773–0.874)	<0.001	0.092	−132.49	3.99	0.005	272.97
**Hemorrhagic transformation**							
Base model III	0.722 (0.637–0.808)	<0.001	Reference	−102.59	Reference	Reference	211.19
Base model III + Nr	0.689 (0.595–0.782)	<0.001	0.792	−102.50	0.009	0.675	213.01
Base model III + Nc	0.720 (0.635–0.805)	<0.001	0.814	−102.35	0.024	0.486	212.70
Base model III + L	0.722 (0.635–0.808)	<0.001	0.727	−102.59	0.000	0.914	213.17
Base model III + NLR	0.721 (0.635–0.807)	<0.001	0.761	−102.48	0.011	0.636	212.96
Base model III + P	0.723 (0.640–0.806)	<0.001	0.942	−102.52	0.007	0.703	213.04
Base model III + PNR	0.704 (0.610–0.797)	<0.001	0.918	−102.04	0.055	0.294	212.08
Base model III + NLR/PLT*100	0.721 (0.635–0.807)	<0.001	0.757	−102.34	0.025	0.499	212.69
Base model III + E	0.717 (0.627–0.806)	<0.001	0.539	−102.40	0.019	0.534	212.80
Base model III + NER	0.717 (0.627–0.806)	<0.001	0.556	−102.05	0.054	0.298	212.10
Base model III + M	0.721 (0.634–0.808)	<0.001	0.815	−102.51	0.008	0.681	213.02
Base model III + MNR	0.724 (0.639–0.808)	<0.001	0.906	−102.35	0.024	0.485	212.70

*Base model I includes age and AF. Base model II includes age and admission NIHSS score. Base model III includes AF and admission NIHSS score. NIHSS, national institute of health stroke scale; mRS, Modified Rankin Scale; HT, hemorrhagic transformation; CI, confidence interval; ROC, the receiver operating characteristics; LR test, Log-rank test; LLR, log-likelihood ratio; AIC, Akaike Information Criterion; Nr, neutrophil ratio; Nc, neutrophil count; L, lymphocyte; NLR, neutrophil-to-lymphocyte ratio; P, platelet; PNR, platelet-to-neutrophil ratio; NLR/PLT, NLR-to-platelet ratio; E, eosinophil; NER, neutrophil-to-eosinophil ratio; M, monocyte; MNR, monocyte-to-neutrophil ratio.*

## Discussion

A flood of ratio indicators in the AIS prognosis of outcome have occurred in recent years, which led to some cases where studies mechanically carried out researches on the ratio of diverse biomarkers, especially peripheral blood cells. They rarely explored the ratio indicator in depth and lacked valid meaning, for the reason that the statistical significance of the ratio indicator is only caused by the numerator or denominator. Moreover, neutrophils are confirmed to be associated with the prognosis of AIS, and numerous related ratio indicators have been put forward. Due to concerns of comprehensiveness, all published ratio indicators consisted of neutrophils and other blood cells to our knowledge were included in this study to compare the predictive value of the neutrophil-related ratios and find the best indicator on the 90-day outcome.

In our study, we found that Nr, Nc, NLR, P, PNR, NLR/PLT, NER, and MNR are able to differentiate the HC and AIS groups after matching by age and sex. It could be noticed that the strong statistical significance of NLR, NER, and MNR resulted from N. Therefore, it is more reasonable to use N directly rather than these multiple indicators to differentiate the HC and AIS groups. Furthermore, it was proved that the poor outcome of AIS patients was inversely correlated with PNR and MNR, and none of the neutrophil-related ratios was correlative with the severity of AIS on admission. In addition, Nr, Nc, NLR, PNR, NLR/PLT, NER, and MNR improved the calibration of base model II (Alba et al., 2017), among which, NER worked best. The reason why we chose the variables with a *p* < 0.10 in model 1 as covariates to add in the multivariate analysis was because the univariate regression analysis did not adjust the confounding factors. In order to take more variables into the equation, it was necessary to loosen the standard moderately and not omit the meaningful variables. Taking what was mentioned above into consideration, we drew our final conclusions. When the neutrophil-related ratios were used alone, a lower PNR was the best indicator to predict a poor 90-day outcome of the AIS patients. NER is the best indicator to improve the calibration of the base model to predict the 90-day outcome, and the higher the NER is, the worse the outcome is.

Acute ischemic stroke can cause immune disorders and trigger systemic inflammation characterized by peripheral leukocytes. In turn, activated leukocytes can exacerbate neuron injury and expand the infarct size through different pathophysiological pathways (Iadecola and Anrather, 2011; Planas, 2018; Semerano et al., 2019). Leukocytes and their subtypes have distinct prognostic roles in AIS. Studies have shown that in AIS patients, an elevated leukocyte level increases the risk of cerebral infarction and is associated with a poor prognosis.

Neutrophils are the first peripheral immune cells to penetrate into ischemic areas (Cao et al., 2020). Activated neutrophils secrete harmful substances and inflammatory mediators, which can aggravate ischemic injury and even induce hemorrhagic transformation. Furthermore, neutrophil extracellular traps (Nets) are considered to be another potential mechanism leading to disruption and hemorrhagic transformation, which may be responsible for the association of neutrophils with adverse outcomes (Martinod and Wagner, 2014). High levels of both the neutrophil counts and neutrophil ratio were associated with an increased risk of new stroke, composite events, and ischemic stroke in patients with a minor ischemic stroke or TIA (Zhu et al., 2018). In a recent study, neutrophil stalling of brain capillaries was proved to contribute to reperfusion failure, which offers promising therapeutic avenues for ischemic stroke (El Amki et al., 2020). However, everything has two sides and also comes with both pros and cons. Although neuroinflammation is often depicted as detrimental, there is a growing evidence that alternatively activated, reparative leukocyte subsets and their products can be deployed to improve neurological outcomes. A new neutrophil subset promotes central nervous system (CNS) neuron survival and axon regeneration (Sas et al., 2020).

After stroke, the number of lymphocytes decrease significantly, and activated lymphocytes can aggravate brain tissue damage by releasing a reduced form of nicotinamide-adenine dinucleotide phosphate (NADPH) oxidase, thus, negatively affecting neuroprotection (Ma et al., 2017). Therefore, lymphocyte reduction is considered to be an internal self-protection mechanism.

Various studies have shown that NLR is closely related to AIS dysfunction, short-term mortality, stroke severity on admission, primary unfavorable functional outcome, recurrent ischemic stroke and post stroke infections, greater risk of symptomatic intracranial hemorrhage, poor 3-month functional outcome, and 3-month mortality in AIS patients undergoing reperfusion treatments, which is consistent with our results (Xue et al., 2017; Kocaturk et al., 2019; Chen et al., 2020; Giede-Jeppe et al., 2020; He et al., 2020; AltinbaŞ et al., 2021; Bi et al., 2021; Ferro et al., 2021; Gong et al., 2021; Hu et al., 2021; Huang et al., 2021; Lin et al., 2021). In a recent study, NLR ≥ 9 was an independent predictor of new in-hospital neurologic complications (Heuschmann et al., 2004). High-grade granulocyte count exacerbates inflammatory responses in ischemic areas, exacerbating brain edema and neuronal death. Low lymphocyte counts maintain the immune response of the body to ischemic areas. As a result, an elevated NLR suggests a stronger inhibition of inflammatory and immune responses. It suggests that the neutrophil-to-lymphocyte ratio is associated in patients with AIS. In a study that also compared the predictive value of ratio indicators, it draws the conclusion that platelet-to-lymphocyte, neutrophil-to-lymphocyte, lymphocyte-to-monocyte ratio, and aspartate-to-alanine aminotransferase ratios are inexpensive, easy, fast, and reproducible parameters that can be used in determining the prediction of carotid artery stenosis (Han et al., 2021).

Platelets are activated after being stimulated by various factors, including inflammation and atherosclerosis. Activated platelets gather at damaged endothelial cell sites and release proinflammatory mediators. In addition, activated platelets are involved in the development of atherosclerosis, which will gradually lead to the rupture of atherosclerotic plaques and trigger ischemic events (Franco et al., 2015). Many studies have elaborated that activated platelets release chemicals associated with leukocyte recruitment and interact with leukocytes and neutrophils to exacerbate inflammation and thrombosis (Ishikawa et al., 2012).

PNR predicts that the cause of death in AIS patients may be because the platelet-leukocyte complex exacerbates ischemia-reperfusion injury (Ritter et al., 2000). When AIS occurs, thrombosis can lead to the excessive depletion of platelets to a decrease in platelet count. Therefore, PNR can comprehensively reflect thrombosis and inflammation. In a reported study, PNR was independently associated with early neurological deterioration, hemorrhagic transformation, delayed neurological deterioration, and poor 3-month outcome of AIS in the IVT group. Lower PNR can predict a worse outcome (Wang et al., 2020a). What is more, PNR level has an accuracy in the 3-month prognosis of acute ischemic cerebral infarction (Jin et al., 2019). Both the PNR on admission and 24-h PNR were independently associated with poor functional outcomes. Compared with the PNR on admission, the 24-h PNR may serve as a more reliable marker for a poor prognosis in ischemic stroke patients receiving IVT (Pan et al., 2020).

PLT, on the one hand, is a proverbial parameter that reflects thrombopoiesis, platelet consumption, and senescence for a constant balance of platelets (Daly, 2011). On the other hand, NLR is a value that can be calculated simply from a differential leukocyte count and is known as an indicator of systemic inflammation. Thus, it is feasible to deduce that multiplying these values would be of significant value to predict the severity of the disease, which led to NLR/PLT being included in this study. In a study, NLR/PLT showed statistically significant results, respectively, both at admission (AUC = 0.697) and after 3 months (AUC = 0.661) (Lim et al., 2019).

Eosinopenia is associated with a high infection rate and poor outcome. The exact mechanisms underlying the relationship between eosinopenia and AIS severity remain unclear (Zhao et al., 2019).

NER represents systemic inflammation. In a published study, it was reported that blood eosinophil levels were reduced under a strong stress response (Mathur and Sachdev, 1958). In patients who are hospitalized for AIS, systemic infections such as pneumonia often develop within the next few days as a complication, which contributes to the stroke severity and in-hospital mortality (Wang et al., 2020b). Systemic bacterial infections lead to an increase in blood neutrophil count and a decrease in blood eosinophil count (Gil et al., 2003). The pathophysiological mechanisms of these changes in AIS are still unknown. However, it is known that an inflammatory response occurs at all stages of AIS (Worthmann et al., 2010). Microglia and astrocytes are activated promptly after the cerebral ischemic event (Wang et al., 2007). This leads to the release of proinflammatory cytokines and chemokines (Wang et al., 2007). These, in turn, lead to the destruction of the blood-brain barrier and the subsequent passage of immune cells into the damaged area, which constitutes an essential mechanism of secondary deterioration (Wang et al., 2007; Jayaraj et al., 2019). Based on the above information, it can be suggested that NER, which represents the peripheral neutrophil count to eosinophil count ratio, can be regarded as a good indicator of increased neutrophil and decreased eosinophil levels in the blood, or in other words, inflammation in AIS (Wang et al., 2020c). Compared to the other predictive indicators, NER not only better reflects the systemic inflammation in AIS, but also has the best statistical significance in predicting the 90-day prognosis. Therefore, we believe that a higher NER was associated with a poor prognosis for AIS at admission, and can be used as a useful prognostic tool in predicting the disease prognosis.

Monocytes, which play a particularly important role in the prognosis after AIS, are recruited to the ischemic region (Gunes, 2020). High levels of both the neutrophil counts and neutrophil ratio were associated with an increased risk of a new stroke, composite events, and ischemic stroke in patients with a minor ischemic stroke or TIA. Therefore, MNR can comprehensively reflect thrombosis and inflammation.

**FIGURE 2:**
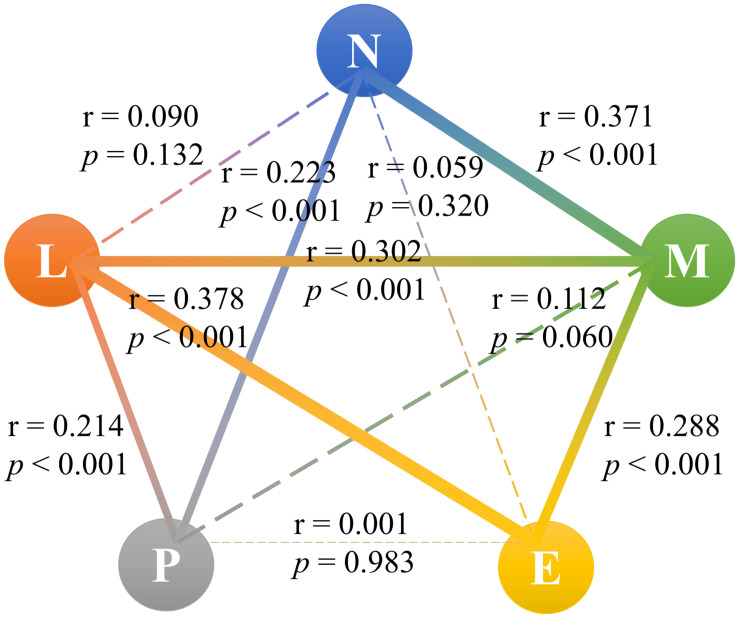
Correlation between neutrophil, lymphocyte, platelet, eosinophil, and monocyte. N, neutrophil; L, lymphocyte; P, platelet; E, eosinophil; M, monocyte.

We also studied the Spearman’s correlation between N, L, P, E, and M and found that between N and P (*r* = 0.223, *p* < 0.001), N and M (*r* = 0.371, *p* < 0.001), L and P (*r* = 0.214, *p* < 0.001), L and E (*r* = 0.378, *p* < 0.001), L and M (*r* = 0.302, *p* < 0.001), and E and M (*r* = 0.288, *p* < 0.001) had weak correlations (0.200 < *r* < 0.390). Other biomarkers presented no direct correlation relationship. Therefore, NLR, PNR, NER, and MNR had independent meanings and did not interfere with each other (Figure 2).

**FIGURE 3:**
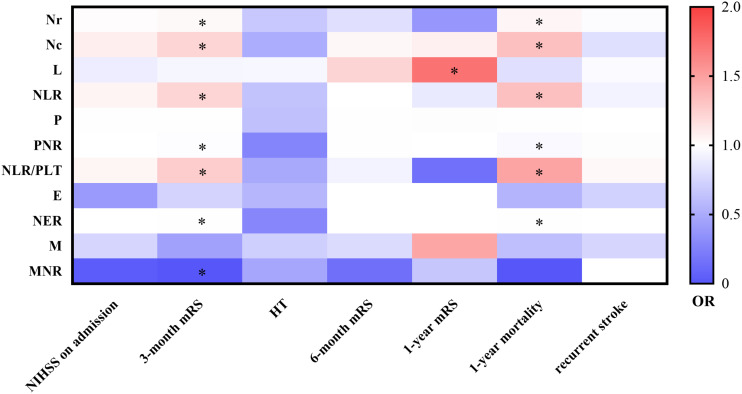
Multivariate logistic regression analyses for prognosis. NIHSS, national institute of health stroke scale; mRS, Modified Rankin Scale; HT, hemorrhagic transformation; OR, odd ratio; Nr, neutrophil ratio; Nc, neutrophil count; L, lymphocyte; NLR, neutrophil-to-lymphocyte ratio; P, platelet; PNR, platelet-to-neutrophil ratio; NLR/PLT, NLR-to-platelet ratio; E, eosinophil; NER, neutrophil-to-eosinophil ratio; M, monocyte; MNR, monocyte-to-neutrophil ratio.

Furthermore, in order to further investigate whether these neutrophil-related ratios have a predictive value for other outcomes, the study was conducted in these 283 patients. Through multivariate logistic analyses, we explored the association between the neutrophil-related ratios and several scales of stroke, including which we had investigated and arrived at the same result, including the NIHSS on admission, 3-month mRS, HT, and which we newly added, including 6-month mRS, 1-year mRS, 1-year mortality, and recurrent stroke. As the heatmap showed, several neutrophil-related ratio levels improved the calibration of clinical prediction models for 1-year mRS and 1-year mortality. Red color stood for risk and OR while blue color means protection. To be noticed, an OR greater than or equal to 2 was shown as the deepest red. One “^∗^” means that a *p*-value was less than 0.05 and more than 0.001. Confounding factors with a *p*-value less than 0.1 in the univariate analysis and also less than 0.05 in the multivariate analysis were adjusted in the second multivariate analysis like model 3 in Table 3 as mentioned previously. It is clearly shown in Figure 3 that the neutrophil-related ratios are more valuable when 3-month mRS and 1 year mortality were observed as prognostic outcomes (Figure 3).

We should be cautious about the indicators of ratio type. On the one hand, they may amplify the function of biomarkers as the numerator or denominator, showing a better prognosis effect. On the other hand, they can also bring about meaningless comparisons between a large number of unnecessary indicators, thus causing trouble to clinical doctors. For a ratio-type indicator to be useful, it should be subjected to several conditions. Firstly, the influence of the numerator and denominator on the prognosis outcome should be opposite. Furthermore, the prognostic effect of the ratio as a whole should outperform the numerator or denominator used alone, and should not be dominated by any of them.

Compared with other studies, our research has the following strengths: all blood samples were taken after admission in 24 h. Also, this study, for the first time, indicated the value of NER in improving the calibration of the base model to predict the worst 90-day outcome which other studies have not yet focused on, and came up with the evidence that a lower PNR can predict a poor 90-day outcome of AIS patients. There are also several limitations to this study. First, the sample size of this study is correspondingly small. Second, due to the limitation of cross-sectional studies, causal conclusions had not been established even when we corrected for the potential confounders in the logistic regression. In addition, there may be a selection bias in our study on account of our patients who are sorted from a single hospital. In the previous study, Post-IVT PNR was proved to be independently associated with early neurological deterioration, hemorrhagic transformation, delayed neurological deterioration, and poor 3-month outcome. Lower PNR can predict a worse outcome. Their data reflected a dynamic change of the PNR value in IVT patients, and based on a large sample size, our results became more reliable and convincing. This study also, for the first time, indicated the value of PNR in predicting the prognosis of IVT. PNR may become a predictive factor in future studies. However, in their study, the baseline PNR value had no association with any of the four outcome measures after the independent sample *t*-test and multivariate logistic regression analysis adjusted for age, sex, current smoking, current drinking, hypertension, diabetes, atrial fibrillation, prior stroke, time from stroke onset to r-tPA infusion, coronary artery disease, systolic blood pressure, diastolic blood pressure, antihypertensive therapy, antiplatelet therapy, antiplatelet therapy, hypoglycemic therapy, baseline blood glucose, and NIHSS score at baseline (Jin et al., 2010). Different from them, we conducted a univariate logistic regression for PNR and found the statistical significance on 90-day mRS. With a multivariate logistic analysis adjusted for age and admission NIHSS scores and this model, which was designed for mRS and was more precise and concise, PNR demonstrated a statistical significance. In another study, both the PNR on admission and the 24-h PNR were independently associated with poor functional outcomes at 3 months. Compared with the PNR on admission, the 24-h PNR may serve as a more reliable marker for a poor prognosis in ischemic stroke patients receiving IVT (Daly, 2011). Our study found that most of the post-thrombolysis neutrophil-related ratios had no significance for the AIS prognosis, suggesting that the neutrophil-related ratios may be potentially time-dependent, which further reflected the significance of a lower PNR, which predicted a poor 90-day outcome of AIS patients and NER, which was the best indicator to improve the calibration of the base model to predict the 90-day outcome.

## Conclusion

Lower PNR was the best indicator to predict a poor 90-day outcome of AIS patients when the neutrophil-related ratios were used alone. NER is the best indicator to improve the calibration of the base model to predict the 90-day outcome, and the higher the NER is, the worse the outcome is.

## Data Availability Statement

The raw data supporting the conclusions of this article will be made available by the authors, without undue reservation.

## Ethics Statement

This study was approved by the Ethics Committee of the Third Affiliated Hospital of Wenzhou Medical University and was carried out in accordance with the Declaration of Helsinki.

## Author Contributions

SZ and GC: conception and design. BG, WP, XH, HH, JR, CY, XZ, TZ, JH, SL, and YG: data acquisition. BG, WP, XH, and HH: data analysis and interpretation. BG, WP, and XH: drafting the article. SZ, GC, BG, WP, XH, HH, JR, CY, XZ, TZ, JH, SL, and YG: critically revising the article for important intellectual content. All authors had full access to all the data in the study and take responsibility for the integrity of the data and accuracy of the data analysis, read and approval the final manuscript.

## Conflict of Interest

The authors declare that the research was conducted in the absence of any commercial or financial relationships that could be construed as a potential conflict of interest.

## References

[B1] Alba A. C. Agoritsas T. Walsh M. Hanna S. Iorio A. Devereaux P. J. (2017). Discrimination and calibration of clinical prediction models: users’ guides to the medical literature. *JAMA* 318 1377–1384. 10.1001/jama.2017.12126 29049590

[B2] AltinbaŞ Ö Demiryürek Ş Işık M. Tanyeli Ö Dereli Y. Görmüş N. (2021). Predictive value of neutrophil-to-lymphocyte, aspartate-to-alanine aminotransferase, lymphocyte-to-monocyte and platelet-to-lymphocyte ratios in severity and side of carotid artery stenosis: are those significant? *Heart Surg. Forum* 24 E072–E078. 33635245 10.1532/hsf.3431

[B3] Bi Y. Shen J. Chen S. C. Chen J. X. Xia Y. P. (2021). Prognostic value of neutrophil to lymphocyte ratio in acute ischemic stroke after reperfusion therapy. *Sci. Rep.* 11:6177. 10.1038/s41598-021-85373-5 PMC7971057 33731740

[B4] Cao X. Zhu Q. Xia X. Yao B. Liang S. Chen Z. (2020). The correlation between novel peripheral blood cell ratios and 90-day mortality in patients with acute ischemic stroke. *PLoS One* 15:e0238312. 10.1371/journal.pone.0238312 32857820 PMC7454963

[B5] Chen C. Gu L. Chen L. Hu W. Feng X. Qiu F. (2020). Neutrophil-to-lymphocyte ratio and platelet-to-lymphocyte ratio as potential predictors of prognosis in acute ischemic stroke. *Front. Neurol.* 11:525621. 10.3389/fneur.2020.525621 33569032 PMC7868420

[B6] Chen Z. Jiang B. Ru X. Sun H. Sun D. Liu X. (2017). Mortality of stroke and its subtypes in China: results from a nationwide population-based survey. *Neuroepidemiology* 48 95–102. 10.1159/000477494 28586776

[B7] Daly M. E. (2011). Determinants of platelet count in humans. *Haematologica* 96 10–13. 10.3324/haematol.2010.035287 21193429 PMC3012758

[B8] El Amki M. Gluck C. Binder N. Middleham W. Wyss M. T. Weiss T. (2020). Neutrophils obstructing brain capillaries are a major cause of no-reflow in ischemic stroke. *Cell Rep.* 33:108260. 10.1016/j.celrep.2020.108260 33053341

[B9] Ferro D. Matias M. Neto J. Dias R. Moreira G. Petersen N. (2021). Neutrophil-to-lymphocyte ratio predicts cerebral edema and clinical worsening early after reperfusion therapy in stroke. *Stroke* 52 859–867. 10.1161/strokeaha.120.032130 33517702

[B10] Franco A. T. Corken A. Ware J. (2015). Platelets at the interface of thrombosis, inflammation, and cancer. *Blood* 126 582–588. 10.1182/blood-2014-08-531582 26109205 PMC4520875

[B11] Giede-Jeppe A. Madzar D. Sembill J. A. Sprugel M. I. Atay S. Hoelter P. (2020). Increased neutrophil-to-lymphocyte ratio is associated with unfavorable functional outcome in acute ischemic stroke. *Neurocrit. Care* 33 97–104. 10.1007/s12028-019-00859-5 31617117

[B12] Gil H. Magy N. Mauny F. Dupond J. (2003). [Value of eosinopenia in inflammatory disorders: an “old” marker revisited]. *La Revue Med. Int.* 24 431–435. 10.1016/s0248-8663(03)00138-3 12829215

[B13] Gong P. Liu Y. Gong Y. Chen G. Zhang X. Wang S. (2021). The association of neutrophil to lymphocyte ratio, platelet to lymphocyte ratio, and lymphocyte to monocyte ratio with post-thrombolysis early neurological outcomes in patients with acute ischemic stroke. *J. Neuroinflammation.* 18:51. 10.1186/s12974-021-02090-6 PMC7896410 33610168

[B14] Gunes M. (2020). Is neutrophil/eosinophil ratio at admission a prognostic marker for in-hospital mortality of acute ischemic stroke? *J. Stroke Cerebrovasc. Dis.* 29:104999. 10.1016/j.jstrokecerebrovasdis.2020.104999 32689649

[B15] Han Y. Li G. Tang Y. Zhang B. Zhan Y. Zhang C. (2021). Effect of rt-PA intravenous thrombolysis on the prognosis of patients with minor ischemic stroke. *Neurol. Res.* 10.1080/01616412.2021.1908672 [Epub ahead of print]. 33847231

[B16] Hankey G. J. (2017). Stroke. *Lancet* 389 641–654. 27637676 10.1016/S0140-6736(16)30962-X

[B17] He L. Wang J. Wang F. Zhang L. Zhang L. Zhao W. (2020). Increased neutrophil-to-lymphocyte ratio predicts the development of post-stroke infections in patients with acute ischemic stroke. *BMC Neurol.* 20:328. 10.1186/s12883-020-01914-x 32873248 PMC7460775

[B18] Heuschmann P. Kolominsky-Rabas P. Misselwitz B. Hermanek P. Leffmann C. Janzen R. (2004). Predictors of in-hospital mortality and attributable risks of death after ischemic stroke: the German Stroke Registers Study Group. *Arch. Int. Med.* 164 1761–1768. 10.1001/archinte.164.16.1761 15364669

[B19] Hu D. Ding C. Jiang X. Xiao J. Li C. Zhang L. (2021). Elevated levels of inflammation markers predict poor outcomes in acute ischemic stroke patients after intravenous thrombolysis. *J. Stroke Cerebrovasc. Dis. Official J. Nat. Stroke Assoc.* 30:105587. 10.1016/j.jstrokecerebrovasdis.2020.105587 33450606

[B20] Huang L. Y. Sun F. R. Yin J. J. Ma Y. H. Li H. Q. Zhong X. L. (2021). Associations of the neutrophil to lymphocyte ratio with intracranial artery stenosis and ischemic stroke. *BMC Neurol.* 21:56. 10.1186/s12883-021-02073-3 33546646 PMC7863476

[B21] Iadecola C. Anrather J. (2011). The immunology of stroke: from mechanisms to translation. *Nat. Med.* 17 796–808. 10.1038/nm.2399 21738161 PMC3137275

[B22] Ishikawa T. Shimizu M. Kohara S. Takizawa S. Kitagawa Y. Takagi S. (2012). Appearance of WBC-platelet complex in acute ischemic stroke, predominantly in atherothrombotic infarction. *J. Atheroscler. Thromb.* 19 494–501. 10.5551/jat.10637 22659534

[B23] Jayaraj R. Azimullah S. Beiram R. Jalal F. Rosenberg G. (2019). Neuroinflammation: friend and foe for ischemic stroke. *J. Neuroinflamm.* 16:142. 10.1186/s12974-019-1516-2 PMC6617684 31291966

[B24] Jin P. Li X. Chen J. Zhang Z. Hu W. Chen L. (2019). Platelet-to-neutrophil ratio is a prognostic marker for 90-days outcome in acute ischemic stroke. *J. Clin. Neurosci. Official J. Neurosurg. Soc. Aust.* 63 110–115. 10.1016/j.jocn.2019.01.028 30737090

[B25] Jin R. Yang G. Li G. (2010). Inflammatory mechanisms in ischemic stroke: role of inflammatory cells. *J. Leukoc. Biol.* 87 779–789. 10.1189/jlb.1109766 20130219 PMC2858674

[B26] Kocaturk O. Besli F. Gungoren F. Kocaturk M. Tanriverdi Z. (2019). The relationship among neutrophil to lymphocyte ratio, stroke territory, and 3-month mortality in patients with acute ischemic stroke. *Neurol. Sci.* 40 139–146. 10.1007/s10072-018-3604-y 30327959

[B27] Lim H. Jeong I. An G. Woo K. Kim K. Kim J. (2019). Early prediction of severity in acute ischemic stroke and transient ischemic attack using platelet parameters and neutrophil-to-lymphocyte ratio. *J. Clin. Laboratory Anal.* 33:e22714. 10.1002/jcla.22714 30411816 PMC6818602

[B28] Lin S. K. Chen P. Y. Chen G. C. Hsu P. J. Hsiao C. L. Yang F. Y. (2021). Association of a high neutrophil-to-lymphocyte ratio with hyperdense artery sign and unfavorable short-term outcomes in patients with acute ischemic stroke. *J. Inflamm. Res.* 14 313–324. 10.2147/jir.s293825 33574692 PMC7872943

[B29] Ma M. W. Wang J. Zhang Q. Wang R. Dhandapani K. M. Vadlamudi R. K. (2017). NADPH oxidase in brain injury and neurodegenerative disorders. *Mol. Neurodegener.* 12:7. 10.1186/s13024-017-0150-7 PMC5240251 28095923

[B30] Martinod K. Wagner D. D. (2014). Thrombosis: tangled up in NETs. *Blood* 123 2768–2776. 10.1182/blood-2013-10-463646 24366358 PMC4007606

[B31] Mathur R. Sachdev J. (1958). Mental stress and eosinophil count. *Indian J. Psychol.* 2 381–386. 13562910

[B32] Pan H. Fu M. Ge W. Zhou C. (2020). The effects of changes in platelet-to-neutrophil ratios 24 hours after intravenous thrombolysis on prognosis in acute ischemic stroke patients. *Clin. Neurol. Neurosurg.* 190:105739. 10.1016/j.clineuro.2020.105739 32105907

[B33] Planas A. M. (2018). Role of immune cells migrating to the ischemic brain. *Stroke* 49 2261–2267. 10.1161/strokeaha.118.021474 30355002

[B34] Ritter L. S. Orozco J. A. Coull B. M. McDonagh P. F. Rosenblum W. I. (2000). Leukocyte accumulation and hemodynamic changes in the cerebral microcirculation during early reperfusion after stroke. *Stroke* 31 1153–1161. 10.1161/01.str.31.5.1153 10797180

[B35] Sas A. R. Carbajal K. S. Jerome A. D. Menon R. Yoon C. Kalinski A. L. (2020). A new neutrophil subset promotes CNS neuron survival and axon regeneration. *Nat. Immunol.* 21 1496–1505. 10.1038/s41590-020-00813-0 33106668 PMC7677206

[B36] Semerano A. Laredo C. Zhao Y. Rudilosso S. Renu A. Llull L. (2019). Leukocytes, collateral circulation, and reperfusion in ischemic stroke patients treated with mechanical thrombectomy. *Stroke* 50 3456–3464. 10.1161/strokeaha.119.026743 31619153

[B37] Wang M. Sun Y. Wang Y. Yan X. Jin H. Sun X. (2020a). Platelet-to-neutrophil ratio after intravenous thrombolysis predicts unfavorable outcomes in acute ischemic stroke. *Curr. Neurovasc. Res.* 17 411–419. 10.2174/1567202617666200517111802 32416675 PMC8493787

[B38] Wang M. Sun Y. Wang Y. Yan X. Jin H. Sun X. (2020b). Platelet-to-neutrophil ratio after intravenous thrombolysis predicts unfavorable outcomes in acute ischemic stroke. *Curr. Neurovasc. Res.* 17 411–419. 32416675 10.2174/1567202617666200517111802 PMC8493787

[B39] Wang M. Sun Y. Wang Y. Yan X. Jin H. Sun X. (2020c). Platelet-to-neutrophil ratio after intravenous thrombolysis predicts unfavorable outcomes in acute ischemic stroke. *Curr. Neurovasc. Res.* 17 411–419. 32416675 10.2174/1567202617666200517111802 PMC8493787

[B40] Wang Q. Tang X. Yenari M. (2007). The inflammatory response in stroke. *J. Neuroimmunol.* 184 53–68. 10.1016/j.jneuroim.2006.11.014 17188755 PMC1868538

[B41] Worthmann H. Tryc A. Deb M. Goldbecker A. Ma Y. Tountopoulou A. (2010). Linking infection and inflammation in acute ischemic stroke. *Ann. N. Y. Acad. Sci.* 1207 116–122. 20955434 10.1111/j.1749-6632.2010.05738.x

[B42] Xue J. Huang W. Chen X. Li Q. Cai Z. Yu T. (2017). Neutrophil-to-Lymphocyte Ratio Is a Prognostic Marker in Acute Ischemic Stroke. *J. Stroke Cerebrovasc. Dis. Official J. Nat. Stroke Assoc.* 26 650–657. 10.1016/j.jstrokecerebrovasdis.2016.11.010 27955949

[B43] Zhao H. M. Qin W. Q. Wang P. J. Wen Z. M. (2019). Eosinopenia is a predictive factor for the severity of acute ischemic stroke. *Neural Regen. Res.* 14 1772–1779. 10.4103/1673-5374.258411 31169195 PMC6585555

[B44] Zhu B. Pan Y. Jing J. Meng X. Zhao X. Liu L. (2018). Neutrophil counts, neutrophil ratio, and new stroke in minor ischemic stroke or TIA. *Neurology* 90 e1870–e1878. 29678934 10.1212/WNL.0000000000005554

